# Thyroid hormone receptor actions on transcription in amphibia: The roles of histone modification and chromatin disruption

**DOI:** 10.1186/2045-3701-2-42

**Published:** 2012-12-20

**Authors:** Yun-Bo Shi, Kazuo Matsuura, Kenta Fujimoto, Luan Wen, Liezhen Fu

**Affiliations:** 1 Section on Molecular Morphogenesis, Program in Cellular Regulation and Metabolism (PCRM), Eunice Kennedy Shriver National Institute of Child Health and Human Development (NICHD), National Institutes of Health (NIH), Bethesda, Maryland, 20892, USA; 2 Division of Gene Structure and Function, Research Center for Genomic Medicine, Saitama Medical University, 1397-1 Yamane, Hidaka-shi, Saitama, 350-1241, Japan

**Keywords:** Transcriptional coactivator, Corepressor, Thyroid hormone receptor, Stem cell, Apoptosis, Metamorphosis, *Xenopus laevis* and *tropicalis*, Histone methylation, Histone acetylation, Nucleosome removal

## Abstract

Thyroid hormone (T3) plays diverse roles in adult organ function and during vertebrate development. The most important stage of mammalian development affected by T3 is the perinatal period when plasma T3 level peaks. Amphibian metamorphosis resembles this mammalian postembryonic period and is absolutely dependent on T3. The ability to easily manipulate this process makes it an ideal model to study the molecular mechanisms governing T3 action during vertebrate development. T3 functions mostly by regulating gene expression through T3 receptors (TRs). Studies in vitro, in cell cultures and reconstituted frog oocyte transcription system have revealed that TRs can both activate and repress gene transcription in a T3-dependent manner and involve chromatin disruption and histone modifications. These changes are accompanied by the recruitment of diverse cofactor complexes. More recently, genetic studies in mouse and frog have provided strong evidence for a role of cofactor complexes in T3 signaling in vivo. Molecular studies on amphibian metamorphosis have also revealed that developmental gene regulation by T3 involves histone modifications and the disruption of chromatin structure at the target genes as evidenced by the loss of core histones, arguing that chromatin remodeling is an important mechanism for gene activation by liganded TR during vertebrate development.

## Introduction

Thyroid hormone (T3) affects numerous biological processes in vertebrates and thyroid diseases are arguably the most prevalent group of metabolic disorders in the world [1-3]. In the adult mammals such as humans, T3 deficiency leads to reduced metabolic rate while both hyperthyroidism and hypothyroidism result in abnormal function of diverse organs and tissues [4-6].

T3 plays a critical role for vertebrate development. T3 deficiency during human development leads to a number of developmental defects, including the formation of a goiter, i.e., a lump in the neck due to enlarged thyroid gland, and cretinism, which is manifested with severe mental deficiency and short stature [7,8]. Similar requirement for T3 is also observed in other vertebrates. The most dramatic T3-regulated developmental process is anuran metamorphosis, when an aquatic tadpole is transformed into a terrestrial frog, as first demonstrated a century ago [9-11]. This process resembles the postembryonic, or perinatal development in mammals when plasma T3 levels also peak [12].

T3 can exert its effects at both the genomic level through nuclear T3 receptors (TRs) and the non-genomic levels. The non-genomic effects of T3 involve the binding of T3 to diverse cellular proteins. Among them include the cell surface integrin αVβ3, better known as a receptor for the extracellular matrix, and a number of cytosolic proteins, which have additional, often enzymatic functions [13-19]. In addition, while TRs are predominantly localized in the nucleus even in the absence of T3, some are present in the cytoplasm. Interestingly, cytosolic TRβ can form a complex with the signaling kinase MAPK, which may be responsible for the rapid activation of MAPK by T3 [19], and unliganded TRβ can interact with phosphatidylinosital 3 kinase (PI3K) to activate this signaling pathway [20,21], suggesting that cytoplasmic TR may mediate some non-genomic effect of T3.

The genomic action of T3, i.e., transcriptional regulation through TR, is believed to be the main function of T3 under most physiological and pathological conditions. There are two TR genes in vertebrates, TRα and TRβ genes, encoding two highly homologous proteins with high affinity binding to T3, that can differ in their tissue and temporal expression [22]. Mutations in TRβ have long been shown to be responsible for the human syndrome “resistance to T3” or RTH [23-25]. Most of these TRβ mutants have reduced or abolished ability to bind to T3. More recently, human patients due to mutated TRα genes were reported, with the mutations causing resistance to T3 but have a different phenotype than patients that have mutations in TRβ [26,27]. The importance of TRs in mediating T3 effects has also been substantiated by a large number of gene knockout and transgenic studies in mice [23,28]. Additionally, the total dependence of amphibian metamorphosis on T3 has allowed us and others to show that TR is both necessary and sufficient to mediate the metamorphic effects of T3 [29-40], demonstrating an essential role of TR in T3 signaling during development.

### Gene regulation by TR

T3 can both activate and repress target gene transcription through TRs. TRs are transcription factors belonging to the nuclear hormone receptor superfamily that also include steroid hormone receptors, 9-cis retinoic acid receptors (RXRs), as well as a number of orphan receptors which lack ligands or whose ligands remain to be identified [2,22,41-43]. Like most other members of this family, TRs bind to specific DNA elements called T3 response elements or TREs and regulate target genes bearing such elements in a ligand-dependent manner. TRs are mostly localized in the nucleus even in the absence of T3 and can bind to TREs both in the presence and absence of T3 to regulate target gene transcription. TRs can function as monomers, homodimers, as well as heterodimers with RXRs. For genes induced by T3, TR/RXR heterodimers bind to TREs in target genes even in the context of chromatin [44]. At the unliganded state, the heterodimers represses the target promoter and when T3 is available, the liganded TR/RXR heterodimers then activate the same promoters [2,41-46]. For genes that are down-regulated by T3, the opposite is true. However, relatively few T3 down-regulated genes have been studied and less is known about how T3 represses these genes. Thus, we will focus here only on T3-induced gene expression.

**Figure 1:**
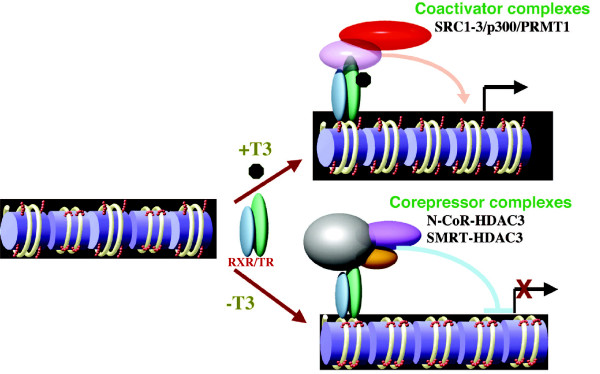
**A model for gene regulation by TR.** In the absence of T3, TR forms heterodimers with RXR (9-cis retinoic acid receptor) and the heterodimer binds to the T3 response elements (TREs) in the target genes to repress their expression by recruiting corepressor complexes such as those containing the related protein N-CoR or SMRT and HDAC-3. When T3 is present, the corepressor complexes are released upon T3 binding to TR, and coactivator complexes such as those containing SRC, p300, and PRMT1, are recruited. SRC and p300 are histone acetyltransferases and PRMT1 is a histone methyltransferase.

TR can recruit corepressor or coactivator complexes to the T3-inducible promoters in the absence or presence of T3, respectively (Figure 1). Many TR-interacting proteins and complexes have been isolated and characterized over the years [2,47-67]. The best-studied TR-corepressors are two highly related proteins N-CoR (nuclear corepressor) and SMRT (silencing mediator of retinoid and thyroid hormone receptors). They form large histone deacetylase (HDAC) 1 or HDAC3-containing complexes, although most evidence suggests that unliganded TR recruits only HDAC3-containing complexes (Table 1) [48-52,54,64,68-77]. Thus, transcriptional repression by unliganded TR likely involves histone deacetylation.

**Table 1:** Known histone modification enzymes involved in gene regulation by Xenopus TR

**Cofactor**	**Histone modification**	**Gene regulation**	**Reference**
HDAC3	Deacetylation	Repression	[68,76,77]
SUV39H1	H3K9 methylation	Repression	[74,75]
G9a	H3K9 methylation	Repression	[73,74]
SRC	Acetylation	Activation	[55,86-88]
P300	Acetylation	Activation	[55,86,89]
CARM1	H3R17 methylation	Activation	[85]
PRMT1	H4R3 methylation	Activation	[84]

T3 binding to TR triggers the release of corepressor complexes and recruitment of coactivator complexes. In vitro and cell culture studies as well as analyses in the reconstituted frog oocytes, where one can study the regulation of chromatinized template, have shown that T3 induces the recruitment of diverse coactivator complexes including ATP-dependent chromatin remodelers, histone acetyltransferase/methyltransferase-containing complexes, as well as TRAP/DRIP/mediator complex (Figure 1) (Table 1) [2,53-60,65-67,78-89], suggesting that gene activation by TR involves histone acetylation and/or methylation as well as chromatin remodeling.

The involvement of these cofactors in T3 signaling during development has been more difficult to substantiate as cofactor knockout mice often have relatively mild phenotypes due to cofactor redundancy or embryonic lethal phenotypes, thus revealing little information about their roles in development. In addition, when gene knockout and transgenesis result in easily identifiable phenotypes, such as mice deficient in N-CoR, p300 (an acetyltransferase), SRC1-3 (steroid receptor coactivator 1, 2, 3, acetyltransferases), or TRAP220 (the TR-binding component of the TRAP complexes) [90-95], the wide involvement of these cofactors in the gene regulation by other transcription factors has made it difficult to link any of the effects directly to T3 signaling defects. Despite these, accumulating evidence has supported roles of some of the cofactors in T3 signaling. One of the earliest was the insensitivity to T3 of the SRC1 knockout mice [96]. More recently, mutations have been introduced to the endogenous N-CoR and SMRT genes in mice and found to cause derepression of T3-inducible genes, supporting their roles in gene repression by unliganded TR [97-99].

The dependence of amphibian metamorphosis on T3 and the ability to manipulate this process have enabled extensive studies on the involvement of cofactors in T3 signaling in vivo. Chromatin immunoprecipitation (ChIP) assays on tadpole tissues have shown that TR recruits corepressor complexes to endogenous T3-inducible genes in premetamorphic *Xenopus laevis* tadpoles when T3 is absent [31,77,100]. More importantly, interfering corepressor function by overexpressing a dominant negative N-CoR that contains only the TR interacting domain of *Xenopus laevis* N-CoR leads to premature upregulation of T3-inducible genes and precocious metamorphosis [101], demonstrating an important role of corepressor recruitment by unliganded TR during development.

When T3 is present either endogenously or by adding it to the tadpole rearing water, the corepressor complexes are released and coactivator complexes, such as those containing histone acetyltransferases SRC3 and p300 and histone methyltransferase PRMT1 (protein arginine methyltransferase 1) are recruited by TR, accompanying the activation of target genes and metamorphosis [84,86-89]. The critical role of coactivators SRC1-3 and p300 in T3 signaling during metamorphosis is supported by transgenic studies. SRCs bind to liganded TR directly and interact with p300 and PRMT1 to form large coactivator complexes. *Xenopus laevis* SRC3 is upregulated during metamorphosis [102] and is recruited to target genes by liganded TR in a gene- and tissue-specific manner during metamorphosis [88]. Furthermore, transgenic overexpression of a dominant negative *Xenopus laevis* SRC3 that contained only the TR-binding domain inhibited both gene activation by T3 and metamorphosis [87], indicating that coactivator recruitment is essential for liganded TR function during metamorphosis.

As the dominant negative SRC3 blocked all coactivator binding to liganded TR, the roles of specific coactivator complexes remained unclear. Interestingly, transgenic overexpression of a dominant negative p300 that contained only the SRC-interacting domain also blocked gene activation and metamorphic changes during either T3-induced or natural metamorphosis [89]. Since this mutant p300 does not interfere with the binding of liganded TR with coactivators directly and only disrupts SRC-p300 interaction, the findings argue that SRC-p300 coactivator complexes or related ones are required for the developmental function of liganded TR. Further support for a role of the SRC-p300 complexes in metamorphosis came from transgenic overexpression of another component of the complexes, PRMT1. Overexpression of wild type PRMT1 enhances TR binding to endogenous target genes, gene activation induced by T3, and the rate of metamorphic progression [84]. Thus corepressor and coactivator complexes play distinct roles in regulating T3-target genes to affect different stages of animal development.

### Chromatin disruption by liganded TR

**Figure 2:**
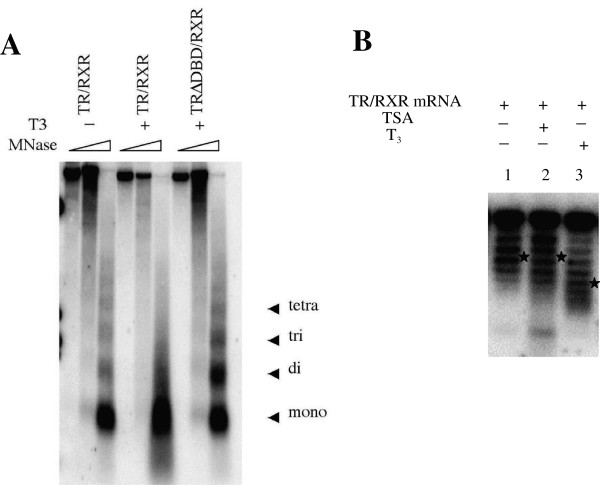
**Transcriptional activation of the HIV-1 LTR by T_3_ but not histone deacetylase inhibitor trichostain A (TSA) leads to chromatin disruption. (A)** Disruption of the chromatin at the HIV LTR by liganded TR requires direct binding of TR to the LTR. *Xenopus* oocytes were injected with the mRNAs encoding RXR and TR or mutant TR lacking the DNA binding domain (TRΔDBD) followed by the injection of a reporter plasmid into the nucleus. The reporter plasmid contained the T3-dependent promoter, the long terminal repeat (LTR), of the human immunodeficiency virus type 1 (HIV-1), directing the transcription of the reporter. The oocytes were treated with T3. After overnight incubation, the reporter plasmid minichromosome was isolated from the oocytes for micrococcal nuclease (MNase) digestion assay with increasing amounts MNase. The digested DNA was purified and analyzed by Southern blot analysis with a labeled LTR probe. **(B)**. DNA topology analysis demonstrates T_3_ but not TSA induces gross alterations of the structure of the LTR minichromosome. The oocytes were injected and treated with T3 or TSA and the LTR plasmid DNA was isolated for supercoiling assay. After electrophoresis on a chloroquine-containing gel to separate the DNA with different number of negative superhelical turns (the higher the negative superhelical turns, the slower the DNA migrated on the gel), the DNA was detected by Southern blot hybridization. Note that the average number of the negative superhelical turns (indicated by a star) was reduced by 2-3 when both TR/RXR and T_3_ are present. As each nucleosome on the circular plasmid generates one negative superhelical turn, the liganded TR induced a structural change equivalent to the loss of 2-3 nucleosomes on the minichromosome. In contrast, TSA had little effect on the number of superhelical turns on the plasmid (compare lanes 2 to 1). See [105,127] for details.

Unlike steroid hormone receptors, TR is predominantly localized in the nucleus even in the absence of T3 and associated with chromatin [103]. Studies in the frog oocyte transcription system, where the exogenous DNA injected into the nucleus is chromatinized, offered the first evidence for direct binding of unliganded TR to a TRE in chromatin [44,104]. Interestingly, when the structure of the minichromosome assembled in the *Xenopus laevis* oocyte was analyzed, it was found that T3 induced the disruption of the ordered nucleosomal organization in the minichromosome in the presence but not in the absence of TR (Figure 2) [44,45,105]. This disruption required DNA binding domain of the TR (Figure 2A), indicating that TR has to bind the TRE to mediate the disruption. By using a supercoiling assay for a circular plasmid, it was shown that the liganded TR-induced chromatin disruption was equivalent to the loss of 2–3 nucleosomes per receptor binding locus (Figure 2B) [45,105]. While the underlying mechanism for the chromatin remodeling remains to be determined, it has been shown that liganded TR recruits chromatin remodeling complexes containing Brg1 and BAF57 to the TRE of the reporter gene [55,78]. Thus, it is likely that such remodeling complexes participate in the removal of the nucleosome near the TRE, thus facilitating the assembly of the transcriptional machinery at the promoter region.

**Figure 3:**
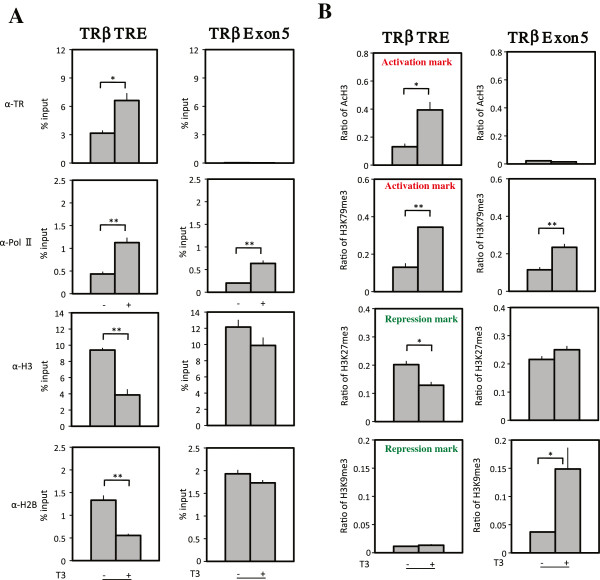
(**A**) **TR binding to the target gene TRβ leads to the recruitment of RNA polymerase α** (**Pol II**) **and loss of core histones during T3induced metamorphosis in *Xenopus tropicalis *intestine.** Tadpoles at stage 54 were treated with or without T3 for 2 days, and the intestine was isolated for ChIP assay with anti-TR, anti-Pol α, anti-H3, or anti-H2B antibody. The immunoprecipitated DNA was analyzed by qPCR for the presence of the TRE region of the TRβ promoter or a region of TRβ exon 5 as a negative control. Note that TR is bound to the promoter but not the exon 5 in the absence of T3 in premetamorphic tadpoles. In the presence of T3, TR binding to the TREs was increased at the promoter, accompanied by the recruitment Pol II and reduction in total histones at the TRE region. Increased Pol II was also observed in the exon region due to increased transcription in the presence of T3. Error bars indicate s.e.m. (n=3). The one and two stars indicate pairs of samples with significant differences, p < 0.05 and p < 0.01, respectively. (**B**). Changes in all histone activation marks but only one of the two repression marks correlate with gene activation by liganded TR. Premetamorphic tadpoles at stage 54 were treated with T3 for 2 days. The intestine was isolated and subjected to ChIP assay with anti-AcH3, anti-H3K79me3, anti-H3K27me3 or anti-H3K9me3 antibody. ChIP signals were normalized with the ChIP signals of histone H3 in (**A**) for the corresponding promoter/exon regions. Error bars indicate s.e.m. (n=3). The one and two stars indicate pairs of samples with significant differences, p < 0.05 and p < 0.01, respectively. See [113] for details.

More recently we have investigated chromatin changes in vivo by using the model system of intestinal remodeling during metamorphosis in *Xenopus tropicalis*, a species highly related to the well-studied *Xenopus laevis*[106,107]. Intestinal remodeling involves the degeneration of larval epithelium and concurrent do novo development of the adult epithelial stem cells, followed by their proliferation and differentiation into the adult epithelium [108-110]. This process resembles the maturation of the mammalian intestine during perinatal development when plasma T3 level is high [111,112]. The simplicity and cell composition of the intestine has made it a valuable model to study the mechanism of TR action during development. By using ChIP assay with antibodies again core histones H2B and H3, we have recently shown that during either T3-induced or natural metamorphosis, T3 induces the removal of core histones from the TRE regions of T3 response genes, including TRβ, in the *Xenopus tropicalis* intestine, accompanying increased TR binding and recruitment of RNA polymerase II (Figure 3A) [113]. This finding suggests local removal of nucleosomes by liganded TR, consistent with the observations in the frog oocyte transcription system. Interestingly, both Brg1 and BAF57, component of the Brg1-containing chromatin remodeling complex, are upregulated or expressed during metamorphosis in *Xenopus laevis*[78], suggesting that the nucleosome removal during intestinal metamorphosis involve the recruitment of this complex by liganded TR.

### T3-induced histone modifications at target genes

The recruitment by TR of a number of cofactor complexes containing histone modification enzymes implicates a role of histone modification in gene regulation by TR. Studies in the frog oocyte showed early on that HDAC activity and chromatin assembly were both required for efficient promoter repression by unliganded TR in vivo [46,114]. ChIP assays with antibodies against acetylated histones showed that T3 treatment or blocking HDAC activity leads to increased histone acetylation at T3-target genes both in mammalian cell cultures and frog oocytes as well as during frog metamorphosis [114-117]. More importantly, treatment of premetamorphic tadpoles with a histone deacetylase inhibitor leads to precocious induction of T3 response genes in the absence of T3, supporting a role of deacetylase in gene repression by unliganded TR in premetamorphic tadpoles [118,119].

In addition to histone acetylation, histone methylation and phosphorylation have also been implicated to play a role in gene regulation by TR in the frog oocyte transcription system [75]. During *Xenopus tropicalis* metamorphosis, gene activation by TR is accompanied by changes in the methylation levels of various histone residues [113,120,121] (Figure 3B). Bilesimo et al. [120] observed that in the brain and tail fin of tadpoles treated with T3, gene activation by T3 was associated with distinct patterns of histone methylations and that gene-specific patterns of TR binding to target genes correlated with gene-specific modifications of H3K4 methylation. Furthermore, treatment of tadpole tail fin with an inhibitor of histone demethylases led to increased T3-response gene expression and TR binding to TREs, supporting a role of histone methylation in gene regulation by T3. In our own studies on intestinal remodeling during metamorphosis, we observed that of the three activation histone methylation marks analyzed, all were increased upon gene activation by T3 (Figure 3B) [113]. In contrast, of the two repression histone methylation marks analyzed, only one was reduced upon gene activation by T3 while the other did not change at the TRE region and was increased in the downstream transcribed region (Figure 3B) [113]. As the activation and repression marks were defined based on correlations with gene expression levels in cultured cells, these findings suggest that tissue and/or developmental context may affect the utilization of histone modification patterns in vivo.

T3 induces changes in histone modifications at target genes presumably by recruiting different cofactor complexes via TRs. ChIP assays have shown that during *Xenopus laevis* or *tropicalis* metamorphosis, increased histone acetylation correlates with the activation of T3 target genes (Figure 3B), the release of corepressors and the recruitment of coactivators [31,77,86-89,100,113,117,120]. Furthermore, transgenic studies have shown that the recruitment of coactivator complexes containing acetyltransferases SRC3 and p300 or related complexes is essential for the histone acetylation, gene activation and metamorphosis [87-89], demonstrating a role of histone acetyltransferase-containing coactivator complexes in T3 signaling during development.

The increase in histone methylation at T3 target genes during metamorphosis is likely due to the recruitment of histone methyltransferases. At least three histone methyltransferases, CARM1 (coactivator-associated arginine methyltransferase 1), PRMT1, and Dot1L (Dot1-like), have been shown to be expressed during metamorphosis in *Xenopus laevis* or *tropicalis*[84,85,122]. Among them, Dot1L is induced by T3 during metamorphosis directly at the transcription level [122], while PRMT1 is indirectly induced by T3 via the induction of c-Myc gene [84,123]. PRMT1 is known to be associated with the SRC-p300 complexes and is recruited to T3 target genes during metamorphosis [84]. More importantly, transgenic overexpression of PRMT1 leads to increased expression of T3 target genes and accelerated metamorphosis [84,124], supporting a role of PRMT1 in T3 signaling. Dot1L is the only known histone methyltransferase capable of methylating H3K79, which is correlated with gene activation by T3 (Figure 3B) [113]. Thus, T3 activates the Dot1L gene, and Dot1L in turn feeds back positively on liganded TR function during metamorphosis by methylating H3K79 at T3 target genes.

## Conclusion

**Figure 4:**
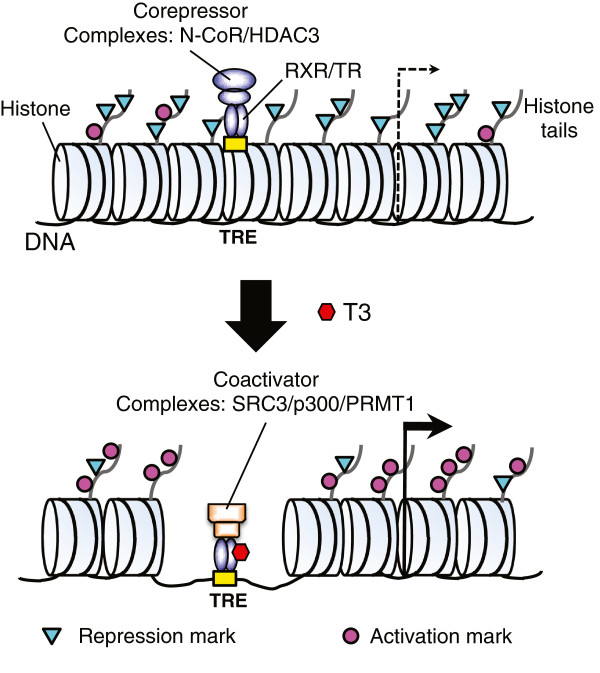
**A model for gene regulation by TR.** T3 functions by regulating gene transcription through T3 receptors (TRs). In the absence of T3, TR/RXR heterodimer binds to TREs in the target genes and recruits corepressor complexes such as the N-CoR-HDAC3 complex. This leads to the establishment of a repressed chromatin state with ordered nucleosomal arrays and abundant repression marks. When T3 is present, TR/RXR recruits coactivator complexes such as those containing SRC, p300, and PRMT1. This leads to the loss of nucleosomes and modifications of the histone tails, leading to gene activation. See [113] for details.

T3 has long been known to be critical for human development, mainly due to its effect on perinatal development. The developmental mechanisms of T3 action, however, have been more difficult to decipher in mammals. Amphibian metamorphosis offers an opportunity to dissect the function and associated mechanisms of T3 action during development without the complication of maternal influences. This has enabled the demonstration of dual function roles of TR during development [33]. More importantly, studies in frogs were the first to provide in vivo evidence for the requirement cofactors in TR function in development. More recent studies in cell cultures, frog oocytes, as well as during metamorphosis, have shown that during vertebrate development, when T3 is absent or at low levels, TR/RXR heterodimers recruit corepressor complexes at T3 inducible genes to establish a repressive chromatin structure in part by using repression histone marks (Figure 4). When T3 becomes available, the corepressor complexes are removed and coactivator complexes are recruited. These complexes help to disrupt the ordered chromatin structure, causing local release of nucleosomes and increases in the levels of activation histone marks (Figure 4). Consequently genes are activated and the developmental effects of T3 are transduced toward downstream events. Clearly, many important questions remain to be addressed. Of immediate interests are the roles of different histone modification enzymes and chromatin remodeling complexes in tissue- and gene-specific regulation of target genes, which is likely critical for tissue specific effects of T3 during development. Are different enzymes recruited by TR in a gene- and tissue-specific manner during development? Do different cofactors affect the utilization and function of other cofactors? While histone modifications have been correlated with gene regulation, are or how are they required for gene regulation by T3 and vertebrate development? The ability to manipulate amphibian metamorphosis will no doubt ensure that this model will continue to serve well the mechanistic studies on TR function in vivo in the foreseeable future, especially in light of the recent advancement in gene knockout studies in *Xenopus*[125,126].

## Competing interests

The authors declare that they have no competing interests.

## Authors’ contributions

All authors participated in the writing of the review. All authors read and approved the final manuscript.
